# Tyrotoxicon in Food

**Published:** 1887-08

**Authors:** 


### TYROTOXICON IN FOOD.

At the meeting of the Michigan State Board of Health, April last, Professor Victor C. Vaughan, made a report on tyrotoxicon, which he has found in poison chesse, milk, ice cream and oysters, and an abstract of this report has been published, by the Board, in advance of the annual report, which will contain the paper in full.

Professor Vaughan has established the identity of tyrotoxicon and diazobenzole, and he thinks that the latter, or some closely allied substance, will be found in all those foods which, from putrefactive changes, produce nausea, vomiting and diarrhœa. He further thinks that while tyrotoxicon is not the only poison which may be developed in milk, and that while decomposed milk is not the only cause of the summer diarrhœas of children, the rational and imperative demar d in such cases in milk-fed children is to stop feeding milk in any form. He recommends as food substitutes, chicken and mutton broths, meat juice, and rice or barley water.

The abstract contains Prof. Vaughan's rules for the prevention of the development of tyrotoxicon in milk. They are very good and thorough, but compliance with them in large cities with present methods and prices is impracticable.

